# Results from Use of Dowd’S Phosphatometer and Tonic

**Published:** 1912-04

**Authors:** Chauncey P. Smith

**Affiliations:** Univ. of Penna. Buffalo


### Results from Use of Dowd's Phosphatometer and Tonic.

Chauncey P. Smith, M. D. (Univ. of Penna.)
Buffalo.

AFTER two years work with Dowd's Phosphatometer, its findings and his remedies to correct pathological conditions presented by its use, I would submit the following conclusions based on thirty cases observed.

For some reason in the analysis of urine—the element phosphorus seems to have been neglected, while various other constituents have been unduly emphasized and enlarged upon although their clinical significance has never been made clear. This is more extraordinary as physiology, chemistry and zoology have definitely shown that of all the elements, phosphorus stands

---

* "A Practical Method for the Control of Typhoid, as applied to the Water Shed of Baltimore. By Marshall Langton Price, M.D., William Royal Stokes, M.D., and C. W. G. Rohrer, M.D., Baltimore. *Journal American Medical Association*, January 20, 1912.

as the keystone in the arch of chemic compounds forming the basis of life. It need only be mentioned that the dominant element of the brain and spinal cord which govern tissues of a lower plane, and of the semen reduced to their ultimate component is phosphorus. In all experimental work for the production of life, biology points towards this all important substance. Remembering these facts which have been known to workers in the sciences of plant and animal life, is it not singular that they should have escaped their relative importance in the science of medicine?

Dowd's Phosphatometer is an instrument whose technique is simple and by means of which the estimation of the amount of phosphorus eliminated can be made in a short time. All urine examined should be subjected to this test as a matter of routine as to my mind it is of greater clinical value in a greater number of cases than the test for sugar.

The clinical cases in which it is of great benefit may be divided by the analysis of the urine into two groups: A. Those with phosphatic excess or waste. B. Those with deficient amount of phosphorus in a body as a whole.

Class A. Those with phosphatic waste. Clinically symptoms complained of are loss of appetite, insomnia, mental unrest, irritability, "temper on edge," "patient carries his business to bed." Does not feel well but cannot give symptoms in an orderly sequence which would point to some disease. The phosphatic index is high, often being 150 plus.

Class B. Those with decreased phosphatic index. Clinical symptoms being loss of energy, cannot "eat" up as much work as formerly, "all in" at 3-5 in the afternoon, tired, sleep does not refresh, vague indefinite pains and accentuation of any ailment or chronic condition to-wit: if patient has headaches they will become more frequent and appear at shorter intervals; if stomach is irritable—it becomes more so—any weak point in the anatomy is brought to the foreground with this condition.

Class A. Clinical Cases. 1. T.G.S. aet. 70. Symptoms chronic gas formation with cardiac oppression in consequence. Attacks have absolutely no relation to character of food or time of eating. Could not walk a block without stopping to regain breath; nitro-glycerine would give relief by causing belching; mental despondency; irritable—would put overwhelming importance on trifles. Phosphatic index 175 plus. Patient was given appropriate remedies for five weeks to decrease phosphatic elimination. At the end of that time the phosphatic index was normal. Symptoms subsided; wife said "patient was better than he had been for five years."

Case 2 Jew aet. 34. Manager of clothing store. Complained of loss of sleep—would get up seven or eight times a night and smoke in effort to quiet himself; was irritable so much that his clerks complained; no appetite; worried; face lined; loss of weight in past six months.

Phosphatic Index 175 plus. Appropriate medication. After a progressive betterment of all symptoms, patient was discharged well in five weeks.

In all these cases that which is very gratifying to a physician, is that one can prognose 30 per cent. improvement in two weeks and get it. Again by analysis of the urine one can accurately speak of patient's condition without information from patient. Many times to test out this, I have asked patient to say nothing until urine was examined when I would say "you are 50 per cent. better" and this statement would be confirmed by the diminution or cessation of the various ailments originally complained of.

Class B. Those with deficient amount of phosphorus in the body as a whole.

Case 1. The most striking and accurate history is that of my own condition. For five weeks I had lassitude, tired at 3 P. M., spent most of the time on the sofa, no ambition, sleep did not refresh; food had no attraction. Finally I saw Dr. Pryor who found I had a temperature of 99.5 degrees. Said no doubt I had been running temperature for a fortnight or more. Was put to bed. Physical examination negative; Widal test negative; urine negative; blood count negative; diagnosis negative; Hydrochloric acid and nux ordered and taken for a week; no marked improvement; still indifferent to food, work, weather, interests. On own initiative bought some of Dowd's tonic as I had heard most extravagant claims of its virtues from him. Sceptically and indifferently I began taking it at 5 P. M. and within an hour felt better. At dinner that night the family—who are close observers—said that I seemed more like myself than I had for some time. Briefly in three days I was myself.

I have used this tonic in a number of cases and when judiciously given immediate and permanent results can be looked for. I have seen and heard of slow healing wounds being quickened; of obstinate corneal ulcers cicatrizing; of coughs that hang on, disappearing.

The evidence to my mind is very clear that in this phosphorus mixture of Dr. Dowd we have a very important addition to our armentarium and particularly in those cases where we used to be at sea as to what to do and chiefly did nothing.

69 CHIPPEWA STREET.

